# Effect of vitamin D3 supplementation on cellular immunity and inflammatory markers in COVID-19 patients admitted to the ICU

**DOI:** 10.1038/s41598-022-22045-y

**Published:** 2022-11-03

**Authors:** Mikhail V. Bychinin, Tatiana V. Klypa, Irina A. Mandel, Gaukhar M. Yusubalieva, Vladimir P. Baklaushev, Nadezhda A. Kolyshkina, Aleksandr V. Troitsky

**Affiliations:** 1 Intensive Care Unit, Federal Research Clinical Center of Specialized Types of Medical Care and Medical Technologies of the Federal Medical and Biological Agency of Russia, 28 Orekhoviy Blvd, Moscow, 115682 Russia; 2 Anesthesiology and Intensive Care Unit, I.M. Sechenov First Moscow State Medical University (Sechenov University), 8-2 Trubetskaya str., Moscow, 119991 Russia

**Keywords:** Lymphocytes, Viral infection, Predictive markers

## Abstract

Vitamin D as an immunomodulator has not been studied in patients with severe COVID-19. This study aimed to estimate the efficacy of vitamin D3 supplementation on cellular immunity and inflammatory markers in patients with COVID-19 admitted to the intensive care unit (ICU). A single-center, double-blind, randomized, placebo-controlled pilot trial was conducted (N = 110). Patients were randomly assigned to receive a weekly oral dose of 60,000 IU of vitamin D3 followed by daily maintenance doses of 5000 IU (n = 55) or placebo (n = 55). Primary outcomes were lymphocyte counts, natural killer (NK) and natural killer T (NKT) cell counts, neutrophil-to-lymphocyte ratio (NLR), and serum levels of inflammatory markers on 7th day of treatment. On day 7, patients in the vitamin D3 group displayed significantly higher NK and NKT cell counts and NLR than those in the placebo group did. The mortality rate (37% vs 50%, P = 0.16), need for mechanical ventilation (63% vs 69%, P = 0.58), incidence of nosocomial infection (60% vs 41%, P = 0.05) did not significantly differ between groups. Vitamin D3 supplementation, compared with placebo, significantly increased lymphocyte counts, but did not translate into reduced mortality in ICU.

Trial Registration: ClinicalTrials.gov Identifier: NCT05092698.

## Introduction

The severe course of coronavirus disease 2019 (COVID-19) manifests itself as a pronounced dysregulation of innate and adaptive immunity, hyperinflammatory reaction, and massive production of proinflammatory cytokines. Generally, patients with severe COVID-19 display lymphopenia, which includes reduced T, natural killer T (NKT), and natural killer (NK) cell counts, sometimes lower B cell counts, and an increased neutrophil-to-lymphocyte ratio (NLR)^1^. NK cells represent a non-specific cellular component of immediate innate immunity and primarily control the acute phase of viremia^2,3^. However, T cell responses are critical for long-term surveillance. NKT cells represent a unique subset that shares some characteristics with both NK and T cells, and is particularly interesting because of their participation in host defenses against many viral infections by contributing to both innate and adaptive immune responses. Decreased lymphocyte levels were found to be a significant predictor of severe illness and mortality in COVID-19 patients^4,5^.

Vitamin D has important immunomodulatory effects owing to its broad spectrum of activities on the metabolism and activity of macrophages, T cells, and B cells^6^. Vitamin D is known to reduce the levels of proinflammatory cytokines and modulate T-cell proliferation. It also stimulates production of various antimicrobial and antiviral peptides^7^. The expression of the vitamin D receptor and vitamin-D3-metabolizing enzymes (CYP27B1, etc.) in monocytes, macrophages, NK cells, NKT cells, B-cells, and T-cells suggests that these immune cells may produce and use the active form of vitamin, 1,25-dihydroxyvitamin D [1,25(OH)2D], to support cellular immunity^8^.

A high prevalence of vitamin D deficiency is associated with mortality in critically ill patients including patients with COVID-19^9,10^.

We investigated whether vitamin D3 could be used as an adjuvant therapy for COVID-19, as this may have immediate clinical and economic implications in the context of the ongoing COVID-19 pandemic. Considering the immunomodulatory properties of vitamin D, we wanted to explore whether increasing serum 25-hydroxyvitamin D [25(OH)D] concentration may slow disease progression and possibly improve the survival rate.

The main hypothesis of this trial (COVID-VIT) was that therapy with cholecalciferol (60,000 IU/weekly) followed by daily 5000 IU may result in higher NK and NKT cell counts, reduce inflammation, and improve the outcome of patients with severe and critical COVID-19.

## Results

Demographics, clinical and biochemical characteristics of both groups are shown in Table 1.

**Table 1:** Baseline demographic, clinical, and biochemical characteristics of the COVID-19 patients admitted to the ICU^1^.

	Vitamin D3 (n = 52)	Placebo (n = 54)	*P* value^2^
Age, years	64.5 (57–71)	63.5 (54–81)	0.92
Age > 70 y. o	14 (27)	21 (39)	0.19
Sex, n (%) male	22 (42)	31 (57)	0.12
SARS-CoV-2 RT-PCR positive, n (%)	46 (88)	45 (83)	0.86
Ground-glass opacity on computed tomography scan (> 50% area), n (%)	38 (73)	42 (78)	0.57
SOFA, score	3 (1–4)	3 (1–3)	0.87
APACHE II, score	12 (7–20)	12 (8–19)	0.94
**Coexisting disease**			
Coronary artery disease, n (%)	23 (44)	17 (32)	0.18
Arterial Hypertension, n (%)	34 (65)	34 (63)	0.79
Diabetes mellitus, n (%)	16 (38)	14 (26)	0.58
Chronic obstructive pulmonary disease, n (%)	3 (7)	3 (5.5)	0.75
Liver disease, n (%)	1 (2)	1 (2)	1.00
Chronic renal disease	1 (2)	2 (3.7)	1.00
Cerebrovascular disease, n (%)	9 (17)	9 (17)	0.61
Cancer, n (%)	3 (5.8)	4 (7.4)	1.00
**Concomitant medication and respiratory support at admission**			
Anti-IL-6 receptor monoclonal antibodies, n (%)	12 (23)	8 (15)	0.28
Glucocorticosteroids, n (%)	30 (58)	26 (48)	0.33
Antibiotics, n (%)	48 (92)	49 (90)	0.77
Anticoagulant, n (%)	50 (96)	53 (98)	0.54
High-flow oxygen therapy, n (%)	43 (83)	46 (85)	0.73
Mechanical ventilation, n (%)	21 (40)	16 (30)	0.25
**Biochemical parameters**			
25(OH)D, ng/mL (N 30–80 ng/mL)	9.6 (5.6–21)	11 (8.6–15)	0.57
White blood cell count, 10^9^/L (N 4.5–9 10^9^/L)	9 (7–12)	8 (6.5–9.5)	0.09
Lymphocytes, 10^9^/L (N 1.5–4.5 10^9^/L)	0.7 (0.54–0.98)	0.9 (0.60–1.1)	0.11
NK cells (CD3−CD56+CD16+), % (N 9.9–22)	1.3 (0.8–2)	1.3 (0.89–2.6)	0.25
NKT cells (CD3+CD56+CD16+), % (N < 10)	0.5 (0.3–1.3)	0.9 (0.2–2.3)	0.63
NLR (N 0.78–3.53)	10.4 (6.9–15.9)	8.5 (5.1–13.7)	0.07
Neutrophils, 10^9^/L (N 2–7.5 10^9^/L)	8.4 (5.5–11.3)	6.8 (5.2–8.6)	0.08
Platelets, 10^9^/L (N 150–380 10^9^/L)	215 (168–298)	207 (168.3–292)	0.61
Creatinine, μM/L (N 53–97 μM/L)	72 (60–108)	80 (64–100)	0.46
Total bilirubin, μM/L (N 3.4–17.1 μM/L)	11 (8–18)	11 (9–15)	0.88
Total calcium level, mM/L (1.9–2.6 mM/L)	2.1 (1.8–2.1)	2.1 (1.9–2.2)	0.89
Ionized calcium level, mM/L (N 1.05–1.37 mM/L )	1.2 (1.10–1.35)	1.2 (1.06–1.25)	0.91
Interleukin-6, pg/mL (N < 7 pg/mL)	190 (97–620)	77 (31–316)	0.25
D-dimer, μg/mL (N < 0.5 μg/mL)	0.9 (0.5–1.8)	0.8 (0.4–2)	0.79
Fibrinogen, g/L (N 2–4 g/L)	4 (3.2–4.9)	4 (3.4–6)	0.15
Procalcitonin, ng/mL (N < 0.05 ng/mL)	0.54 (0.24–2)	0.3 (0.13–0.8)	0.04
C-reactive protein, mg/L (N < 5 mg/L)	194 (92–278)	125 (85–194)	0.04

^1^Numerical data are expressed as median (IQR); categorical data are shown as the number of cases (percentage).

^2^P values were calculated by Mann–Whitney *U* test, χ^2^ test, or Fisher’s exact test, as appropriate.

^3^N, reference range.

*SARS-CoV-2 RT-PCR positive* positive reverse transcription polymerase chain reaction for the Severe Acute Respiratory Syndrome Coronavirus 2, *SOFA* Sequential Organ Failure Assessment, *APACHE II* Acute Physiology and Chronic Health Evaluation II, *NLR* neutrophil to lymphocyte ratio, *25*(*OH*)*D* serum 25-hydroxyvitamin D.

The time from symptom onset to ICU hospitalization was 9 (IQR 6–12) days. The patients were allocated to one of the groups within 24 h of admission to the ICU. The median age of participants was 64 (IQR 57–77) years.

There were no statistically significant differences between the two groups with respect to demographic and clinical characteristics (Table 1). A Cox regression analysis was performed to adjust the model by possible confounding variables for in-hospital mortality in patients in the vitamin D3 treatment group vs placebo group. HR for coronary artery disease 1.95; 95% CI 1.04, 3.63; P = 0.03. No differences between the vitamin D3 and placebo groups were observed in mechanical ventilation frequency (21 (40%) vs 16 (30%), P = 0.25) and high flow oxygen therapy (43 (83%) vs 46 (85%), P = 0.73) at ICU admission (Table 1). Laboratory tests, including those for white blood cell count, neutrophils, lymphocytes, platelets, NK cells, NKT cells, total bilirubin, creatinine, D-dimer, fibrinogen, and IL-6, did not differ between the two groups, except for procalcitonin level (P = 0.04) and CRP (P = 0.04) (Table 1).

The baseline mean serum 25(OH)D concentration was 13.1 (SD, 9.3) ng/mL; median, 10.8 (6.8–15.8) ng/mL. Forty eight (51%) out of 106 patients had severe vitamin D deficiency (less than 10 ng/mL). There were no between-group differences in serum 25 (OH) D concentrations (9.6 [5.6–20.6] ng/mL in the vitamin D3 group vs 11.2 [8.6–14.9] ng/mL in the placebo group; P = 0.57) at ICU admission. The cumulative dose of vitamin D3 was 185,000 IU (95,000–275,000 IU).

### Primary outcome

On day 7, patients in the vitamin D3 group displayed significantly higher NK and NKT cell counts, as well as a higher NLR, than those in the placebo group (Table 2). No statistically significant differences were observed for other parameters; however, lymphocyte counts and CRP and procalcitonin levels tended to be lower in the vitamin D3 group.

**Table 2:** Dynamics of lymphocyte counts and inflammatory markers in the vitamin D3 and placebo groups before the treatment and on day 7 after the treatment^1^.

Variable	Vitamin D3 (n = 52 «before»; n = 38 «after»)		*P* value^2^	Placebo (n = 54 «before»; n = 18 «after»)		*P* value^2^	*P* value^3^
	Before	After		Before	After		
Lymphocytes, 10^9^/L	0.7 (0.54–0.98)	0.78 (0.42–1.22)	**0.01**	0.9 (0.6–1.1)	1.2 (0.81–1.53)	0.73	0.05
NK cells CD3−CD56+CD16+, %	1.26 (0.8–2)	9.76 (0.9–20)	**0.001**	1.3 (0.9–2.6)	0.92 (0.57–11)	0.39	**0.03**
NKT cells CD3+CD56+CD16+, %	0.5 (0.3–1.3)	2.6 (0.76–12)	**0.001**	0.9 (0.17–2.3)	0.45 (0.07–0.8)	0.15	**0.001**
NLR	10.4 (7–16)	12.5 (7–28)	0.33	8.5 (5–14)	8.3 (5–12)	0.35	**0.01**
Interleukin-6, pg/mL	190 (97–620)	312 (114–2156)	0.38	77 (30–316)	275 (49–4666)	1.00	0.9
C-reactive protein, mg/L	194 (92–278)	85 (13–200)	**0.002**	125 (85–194)	138 (62–247)	0.76	0.07
Procalcitonin, ng/mL	0.54 (0.24–2)	1.1 (0.32–1.9)	**0.02**	0.3 (0.13–0.8)	1.4 (0.4–12)	**0.009**	0.32

^1^Numerical data are expressed as median (IQR); categorical data are shown as the number of cases (percentage).

^2^P values were calculated by Wilcoxon test, χ^2^ test, or Fisher’s exact test, as appropriate.

^3^P values were calculated by Mann–Whitney test between vitamin D3 and placebo groups on day 7.

*NLR* neutrophil to lymphocyte ratio.

Significant values are in bold.

Analysis of laboratory tests before and after treatment indicated that in the vitamin D3 group, unlike in the placebo group, statistically significant increases in lymphocyte, NK, and NKT cell counts, as well as reduced CRP levels were observed (Table 2). Both groups displayed statistically significant increases in procalcitonin levels following treatment, with stronger fold change in the placebo (up to 483%) and vitamin D3 (up to 207%) groups.

The median 25 (OH) D level significantly increased from baseline after treatment with vitamin D3 (from 9.6 (5.6–20.6) ng/mL to 20.6 (11.8–24.8) ng/mL) vs placebo (from 11.2 (8.6–14.9) ng/mL to 10.4 (5.8–12.2) ng/mL). The status of vitamin D on the 7th day was as follows: in the vitamin D3 group, 19 patients with sufficiency, 12—insufficiency, 7—deficiency; in the placebo group, 2 patients with sufficiency, 11—with insufficiency, 5—with deficiency.

### Secondary outcomes

Secondary outcome results are reported in Table 3.

**Table 3:** Secondary outcomes of the COVID-VIT trial^1^.

	Vitamin D3 (n = 52)	Placebo (n = 54)	*P* value^2^
Mortality, n (%)	19 (37)	27 (50)	0.16
LOS ICU, days	15.5 (8–22)	8 (2–15)	0.001
Hospital stay, days	20.5 (15–33)	14.5 (10–23)	0.007
Mechanical ventilation requirement, n (%)	33 (63)	37 (69)	0.58
Mechanical ventilation, days	15 (11–19)	10 (4–16.5)	0.02
Norepinephrine use, n (%)	36 (69)	35 (69)	0.63
Incidence of nosocomial infection, n (%)	31 (60)	22 (41)	0.05
Positive blood culture, n (%)	23 (44)	13 (24)	0.03

^1^Numerical data are expressed as median (IQR); categorical data as number of cases (percentage).

^2^P values were calculated by Mann–Whitney *U* test, χ^2^ test, or Fisher’s exact test, as appropriate. *LOS ICU* length of stay in the intensive care unit; ^3^N is reference range.

Mortality was 37% in the vitamin D3 group (19 of 52 patients) and 50% in the placebo group (27 of 54 patients), P = 0.23), OR 0.576; 95% CI 0.265, 1.252; P = 0.18. Length of stay in the ICU in the vitamin D3 group was statistically significantly different from the placebo group: 15.5 (8–22) days for the vitamin D3 group vs 8 (2–15.3) days for the placebo group, P = 0.001. The same was true for the length of hospital stay: 20.5 (14.8–33) days for the vitamin D3 group vs 14.5 (10–23) days for the placebo group, P = 0.007.

Compared to placebo group patients, vitamin D3 group patients did not appear to significantly differ in terms of the requirement for the use of vasopressors (36 (69%) vs 35 (69%), P = 0.63) and need for mechanical ventilation (33 (63%) vs 37 (69%), P = 0.58), yet the vitamin D3 group spent more days on mechanical ventilation (15 (11–19) days vs 10 (4–16.5) days, P = 0.02). Likewise, there were no significant differences between the vitamin D3 group and the placebo group for incidence of nosocomial infection (60% vs 41%; P = 0.05). However, among the patients in the vitamin D3 group, 44% had positive blood cultures compared with 24% in the placebo group, P = 0.03.

According to Cox regression model, independent risk factors associated with mortality included older age (HR 3.1; 95% CI 1.47, 6.46; P = 0.003), history of oncological disease (HR 9.349; 95% CI 2.438, 35.859; P = 0.001), and the use of vasopressors (HR 6.37; 95% CI 1.4, 29; P = 0.02). A negative blood culture (HR 0.48; 95% CI 0.22, 0.94; P = 0.03) was independently associated with improved survival.

**Figure 1:**
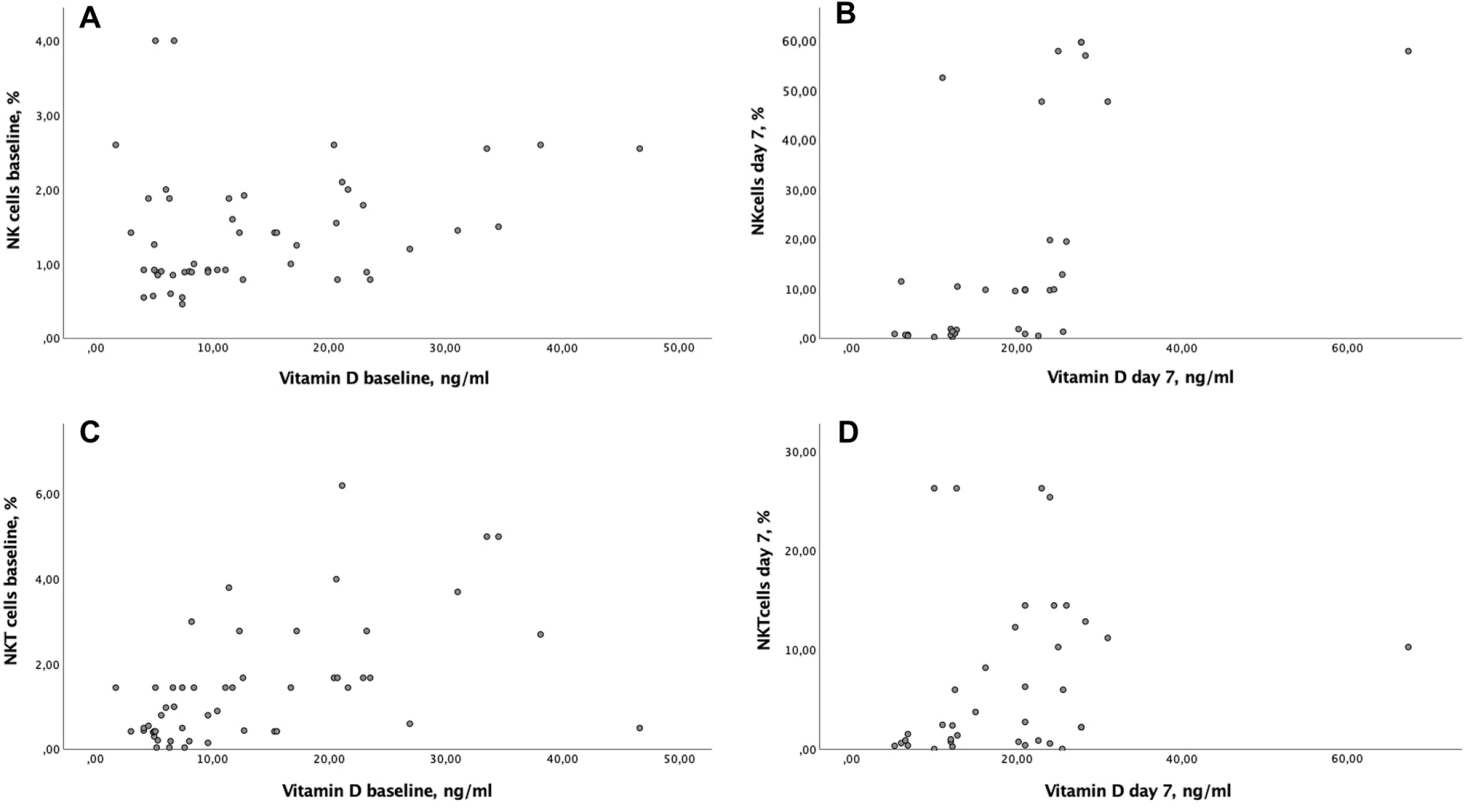
Correlation analysis between the vitamin D and NK/NKT subpopulations of immune cells on admission and on day 7 of stay in the ICU. Baseline levels of vitamin D were positively correlated with NKT cell counts (r = 0.6 (95% CI 0.38; 0.76), P = 0.001), in contrast to NK cells lacking such association (r = 0.26, (95% CI − 0.26; 0.51), P = 0.07) (**a**,**b**). On day 7 of treatment, vitamin D levels were positively correlated with NK cell (r = 0.67 (95% CI 0.42; 0.82), P = 0.001) and NKT cell (r = 0.41 (95% CI 0.08; 0.65), P = 0.013) counts (**c**,**d**).

On admission, baseline levels of vitamin D were positively correlated with NKT cell counts (r = 0.6; 95% CI 0.38, 0.76; P = 0.001), in contrast to NK cells lacking such association (r = 0.26; 95% CI 0.26, 0.5; P = 0.06) (Fig. 1a,b). On day 7 of treatment, vitamin D levels were positively correlated with NK cell (r = 0.67; 95% CI 0.42, 0.82; P = 0.001) and NKT cell (r = 0.41; 95% CI 0.08, 0.65; P = 0.01) counts (Fig. 1c,d). In addition, a positive correlation between initial vitamin D deficiency (less than 9.9 ng/mL) and NKT cell counts on day 7 (r = 0.49; 95% CI 0.13, 0.73; P = 0.008), and a positive correlation between vitamin D insufficiency (10–20 ng/mL) and NK cell counts at baseline was observed (r = 0.39; 95% CI 0.07, 0.64; P = 0.02).

### Adverse events

No hypercalcemia was observed in either group throughout the trial.

## Discussion

In this randomized, double-blind, placebo-controlled trial, administering cholecalciferol to patients with severe or critical COVID-19 within 24 h of ICU admission resulted in a statistically significant increase in serum 25(OH)D concentration, lymphocyte count, NK and NKT cell counts, and reduced CRP levels. A statistically significant increase in procalcitonin levels on day 7 after treatment was observed in both patient groups, with a stronger increase in the placebo group. Nosocomial infections and mortality rates were similar between the study groups. In contrast to the vitamin D3 group, patients who received placebo spent less time in the ICU and hospital, and were less likely to be positive for blood cultures.

The use of vitamin D3 as an adjuvant therapy for COVID-19 was proposed early in the pandemic. This is largely due to its known positive effects on innate and adaptive immunity^6,11^, antiviral properties^12^, stimulated production of anti-inflammatory cytokines, and downregulation of proinflammatory molecules^13^, as well as its well-established protective role in severe lung damage and sepsis^7^.

To our knowledge, this study is one of the first randomized trials to explore the efficacy of vitamin D3 in critically ill ICU-admitted patients with COVID-19. At the time of writing, six randomized clinical trials studying vitamin D and its active forms in COVID-19 patients have been published^14–19^. These included non-ICU patients with moderate and severe COVID-19^14–17^, and mild/asymptomatic COVID-19^18,19^. A meta-analysis of randomized controlled trials found that COVID-19 patients supplemented with vitamin D were more likely to demonstrate lower rates of ICU admission, mortality events, and RT-PCR positivity^20^. However, no statistical significance was observed in ICU outcomes and deaths.

One of the major findings of our study was that vitamin D3 supplementation led to a statistically significant increase in NK and NKT cell counts, with the NK cell subpopulation almost reaching normal values, unlike in the placebo patients, where NK and NKT cells continued to contract. Literature exploring the effects of vitamin D3 on cellular immunity is limited, and no such studies are available for COVID-19. In several studies, including patients with diseases that affect vitamin D metabolism, such as chronic renal failure and vitamin D-resistant rickets, impaired NK cell activity was found^21,22^. In these patients, vitamin D3 supplementation improved and even normalized the NK cell activity. One explanation for this phenomenon may be the positive effect of vitamin D3 on apoptosis of immune cells^23^. Other studies are largely based on in vitro or preclinical animal models^24^ and have somewhat conflicting results^25–27^. Further studies are needed to investigate the effect of vitamin D3 supplementation on NKT and NK cells.

Our trial participants had critically low NK and NKT cell counts upon ICU admission. NK and NKT lymphopenia has been described in COVID-19 patients previously^28,29^, but was significantly more pronounced in our patients. The exact mechanism by which SARS-CoV-2 attenuates circulating NK and NKT cells in patients with disease remains unclear. One possible explanation may be that NK and NKT cells are redistributed to infected tissues^30^. NK cells may migrate into pulmonary tissues in response to inflammatory cytokines released by infected epithelial cells or engaged macrophages^31^. This NK/NKT cell redistribution between peripheral blood and tissues becomes even more pronounced as the COVID-19 course worsens.

Kalicińska et al.^28^ found drastically reduced levels of NK cells and profound dysfunction of T lymphocytes and NK cells in critically ill COVID-19 patients compared to non-ICU COVID-19 patients. Zingaropoli et al.^29^ reported that COVID-19 patients had low NK and NKT cell counts compared to healthy donors. Moreover, a low percentage of NKT cells was independently associated with the severity of the disease and positively correlated with the PaO_2_/FiO_2_ ratio. A correlation between the degree of cytopenia and COVID-19 severity was similarly observed in our study, which focused on ICU-admitted patients with severe and critical COVID-19.

Our analysis demonstrated that NKT lymphopenia is strongly associated with the degree of vitamin D insufficiency, which agrees well with the data on NK cell lymphopenia reported by Vassiliou et al.^32^. The authors demonstrated that vitamin D deficiency was associated with reduced NK cell counts; specifically, vitamin D-deficient patients presented with mild NK lymphopenia (< 100 cells/μL), whereas vitamin D3 insufficient patients had normal NK cell counts (≥ 100 cells/μL).

In our trial, two groups with comparable demographics and accompanying pathologies were formed following randomization. The vitamin D3 group had higher baseline CRP, and procalcitonin levels (P < 0.05). Considering these differences, patients in the vitamin D3 group had an overall higher risk of unfavorable outcomes. Nonetheless, mortality and nosocomial infection rates were similar between the groups. Patients in the vitamin D3 group tended to have more positive blood cultures and spent more time in the ICU and hospital. It is plausible to suggest that patients with severe COVID-19 supplementation with vitamin D3 may lead to improved outcomes, albeit at the expense of longer ICU and hospital stays.

The efficacy of vitamin D3 supplementation in the ICU setting may be limited due to patients' critical illness, as well as due to the therapy they receive. Critically ill patients are known to have a profound decline in 25(OH)D concentration due to attenuated vitamin D metabolism, downregulation of vitamin D-binding protein and albumin production, and compromised conversion of 25(OH)D to 1,25(OH)2D in the kidneys^33^. Intensive therapy for COVID-19 patients includes fluid infusion, extracorporeal membrane oxygenation, and plasma exchange, all of which may significantly reduce vitamin D levels^34^. Considering this feature of vitamin D metabolism in critically ill COVID-19 patients with mind, it becomes clear that a significantly longer time may be needed for vitamin D3 supplementation to have a positive effect, which is mediated by target cell activation and genome expression changes. The use of the active form of vitamin D3 in patients with severe COVID-19 may offer some advantages over cholecalciferol, as calcitriol does not need to be 25-hydroxylated in the liver and shows better absorption in the intestine upon oral consumption^35^. Maghbooli et al.^36^ described the therapeutic benefit of rapid increase the circulating serum 25(OH)D3 levels following oral administration of 25(OH)D3 (Calcifediol, the precursor for active form of vitamin D3) in patients with COVID-19. The authors showed an improvement of immune function by increasing the lymphocytes levels in the blood and an insignificant decreasing the ICU and hospital stay, need for mechanical ventilation, and mortality compared to the placebo. Therefore, interventional trials are needed to uncover possible differences between calcitriol/calcitriol and cholecalciferol supplementation in critically ill COVID-19 patients.

Published reports exploring the efficacy of cholecalciferol in COVID-19 patients^16^ and ICU patients without COVID-19^37^ have used a single, very high dose of cholecalciferol. No improvement in the clinical outcomes was observed. Somewhat counterintuitively, a large single dose of vitamin D3 may be less effective than the regular (daily or weekly) intake of smaller doses. Bolus delivery of vitamin D3 may affect vitamin D metabolism within the target tissue, leading to increased 24-hydroxylation^38^. Therefore, these effects may compromise the ability of 25(OH)D to support the immune response against respiratory infection^12^.

Our study had several limitations. First, it was a single-center study. Second, the results may have been partially affected by the intrinsic heterogeneity of the sample, as our patients had multiple comorbidities. Third, the patients were given a dose of vitamin D3 after a relatively long time from symptom onset to randomization, when they already showed signs of an excessive inflammatory response and organ dysfunction. The early (5–7 days after diagnosis) use of immunological intervention including vitamin D3 in the evaluation of patients with multiple organ dysfunction syndrome may reduce mortality in the most severe patients^39^. Fourth, we did not measure vitamin D-binding protein, free 25(OH)D, or 1,25(OH)2D levels, which could all contribute to the ultimate vitamin D status. However, the quantification of these molecules is complex, and there is no consensus on whether such tests are required. 25(OH)D is currently the best marker for overall vitamin D status and remains the most commonly measured biomarker in clinical medicine.

## Conclusion

Vitamin D3 supplementation, compared to placebo, in patients with severe and critical COVID-19 at a dose of 60,000 U/week followed by 5000 U/day results in a statistically significant increase in NK and NKT cell counts and a reduction in CRP levels. However, this intervention did not translate into reduced mortality or other improvements in ICU outcomes.

## Material and methods

### Study design and participants

This was a prospective, single-center, randomized, placebo-controlled pilot trial conducted at the Federal Scientific and Clinical Center of Specialized Types of Medical Care and Medical Technologies of the Federal Medical and Biological Agency of Russia, which was a designated hospital for COVID-19 patients during the first wave of the pandemic.

### Study approval

The study protocol was approved by the Local Ethics Committee of Clinical Center (protocol № 4 dated 28/04/2020) and registered at http://www.clinicaltrials.gov under NCT05092698; the first registration date is 25/10/2021. In compliance with the national and European Union policies, as well as with the principles of the Declaration of Helsinki, written informed consent was obtained from each patient or the patient’s legal representative. When this was not possible (patients on mechanical ventilation, coma, etc.), the institutional ethical committee, similar to other states of the European Union, approved the use of a “surrogate consent.” Written informed consent was obtained at a later time point, when the patient had survived and regained mental capacity.

#### Inclusion criteria

(1) adults aged ≥ 18 years old admitted to the Intensive Care Unit (ICU); (2) laboratory confirmed COVID-19 (reverse transcription polymerase chain reaction (RT-PCR) for the severe acute respiratory syndrome coronavirus 2 (SARS-CoV-2) from nasopharyngeal swabs) and/or characteristic clinical presentation with computed tomography scan findings compatible with the disease (bilateral multifocal ground-glass opacities ≥ 50%); (3) the serum 25(OH)D below 30 ng/mL.

#### Exclusion criteria

(1) participation in another clinical trial; (2) recent vitamin D3 intake (> 2000 IU/d over 3 months); (3) severely impaired gastrointestinal function; (4) renal insufficiency with creatinine levels above 200 μM/L or requiring renal replacement therapy; (5) hypercalcemia (total calcium > 2.65 mM/L or ionized serum calcium > 1.35 mM/L); (6) pregnancy or breast-feeding; (7) allergy or individual intolerance of the drug or its components; (8) tuberculosis, sarcoidosis; (9) patients within 48 h of their discharge or death.

### Randomization and study interventions

Patients who met the study inclusion criteria were randomly assigned to the vitamin D3 treatment or placebo group (1:1). Random group allocation was performed using computer-generated code managed by an independent researcher who was not involved in the study. The participant’s names was matched with the random number sequence. The names of participants from intervention and placebo was printed and pasted on to the bottles filled with either vitamin D3 or placebo. The bottles used were identical for both active intervention and placebo, and allocation sequence data was kept by independent researcher in a secure place so that it cannot be accessed or influenced by anyone, including the researchers.

Patients in the vitamin D3 group received cholecalciferol orally or via feeding tube at a dose of 60,000 IU once per seven days dissolved in 15 mL of sunflower oil, followed by daily maintenance doses of 5000 IU dissolved in 10 mL of sunflower oil. The maintenance doses begin the day after the intake of the high dose. The high dose was repeated on day 8, 16, 24, 32. Vitamin D3 therapy was administered until the patient was discharge from the ICU or death. The vitamin D3 dosage was chosen based on the recommended dose for patients with vitamin D deficiency^40^. In the placebo group, patients received 15 mL sunflower oil once a week and 10 mL sunflower oil daily. The vitamin D3 and placebo preparations were indistinguishable in terms of visual appearance, taste, smell, texture, or packing. Both preparations were produced by the local hospital pharmacy department and labeled by an associate who was not involved in the trial.

### Outcome measures

#### Primary outcomes

Lymphocyte counts, NK and NKT cell counts in peripheral blood, NLR ratio, serum levels of C-reactive protein (CRP), procalcitonin, interleukin-6 (IL6) and 25(OH)D on day 7 of treatment.

#### Secondary outcomes

ICU mortality, length of ICU and hospital stay, need for mechanical ventilation, use of vasopressors, incidence of nosocomial infections.

### Laboratory assays and data analysis

Blood samples were collected within 24 h of admission for routine laboratory tests, such as blood count, coagulation profile, and serum biochemical tests (including renal and liver function), in the onsite laboratory. Plasma D-dimer concentrations were determined using an ACL TOP 700 automatic coagulation analyzer (Instrumentation Laboratory, CTS Family, Bedford, MA, USA) on a latex-enhanced photometric immunoassay. The plasma fibrinogen concentration was measured according to the photo-optical Clauss method with an ACL TOP 700 automatic coagulation analyzer (Instrumentation Laboratory, CTS Family, Bedford, MA, USA). Serum CRP level was processed by the immunoturbidimetric method using an Architect c800 platform. The serum concentrations of procalcitonin and IL-6 were analyzed using electrochemiluminescence on a Cobas e411 analyzer (Roche Diagnostics GmbH, Mannheim, Germany). Hematological analysis, including blood count, was performed using the flow cytometry method on an ADVIA 2120i (Siemens Healthineers, Erlangen, Germany). Immune cell phenotyping was performed using flow cytometry (ACEA Novocyte Flow Cytometer, ACEA Bioscience, San Diego, CA, USA). Serum 25(OH)D concentrations were assessed by chemiluminescence immunoassay using an ARCHITECT i 2000 SR instrument (Abbott Laboratories, Illinois, USA). The laboratory reference range was 5.0–160.0 ng/mL. All measurements were conducted within 2 h of blood sampling. Vitamin D status was categorized using cutoffs based on serum 25(OH)D concentrations: < 10.0 ng/mL represented severe deficiency, 10.0–19.9 ng/mL represented a deficiency, 20.0–29.9 ng/mL represented insufficiency, and ≥ 30.0 ng/mL represented sufficient 25(OH)D concentrations^41^ All measurements were conducted within 2 h of blood sampling.

Blood microbiological specimens were processed using standard culture techniques that had been validated using BACTEC^®^ 9120 (BD Diagnostics, Sparks, MD, USA). Positive blood culture was defined as growth of a pathogenic bacterial species in ≥ 1 blood culture bottle. A false positive (or contamination) is defined as growth of bacteria in the blood culture bottle that were not present in the patient’s bloodstream, and were most likely introduced during sample collection.

**Figure 2:**
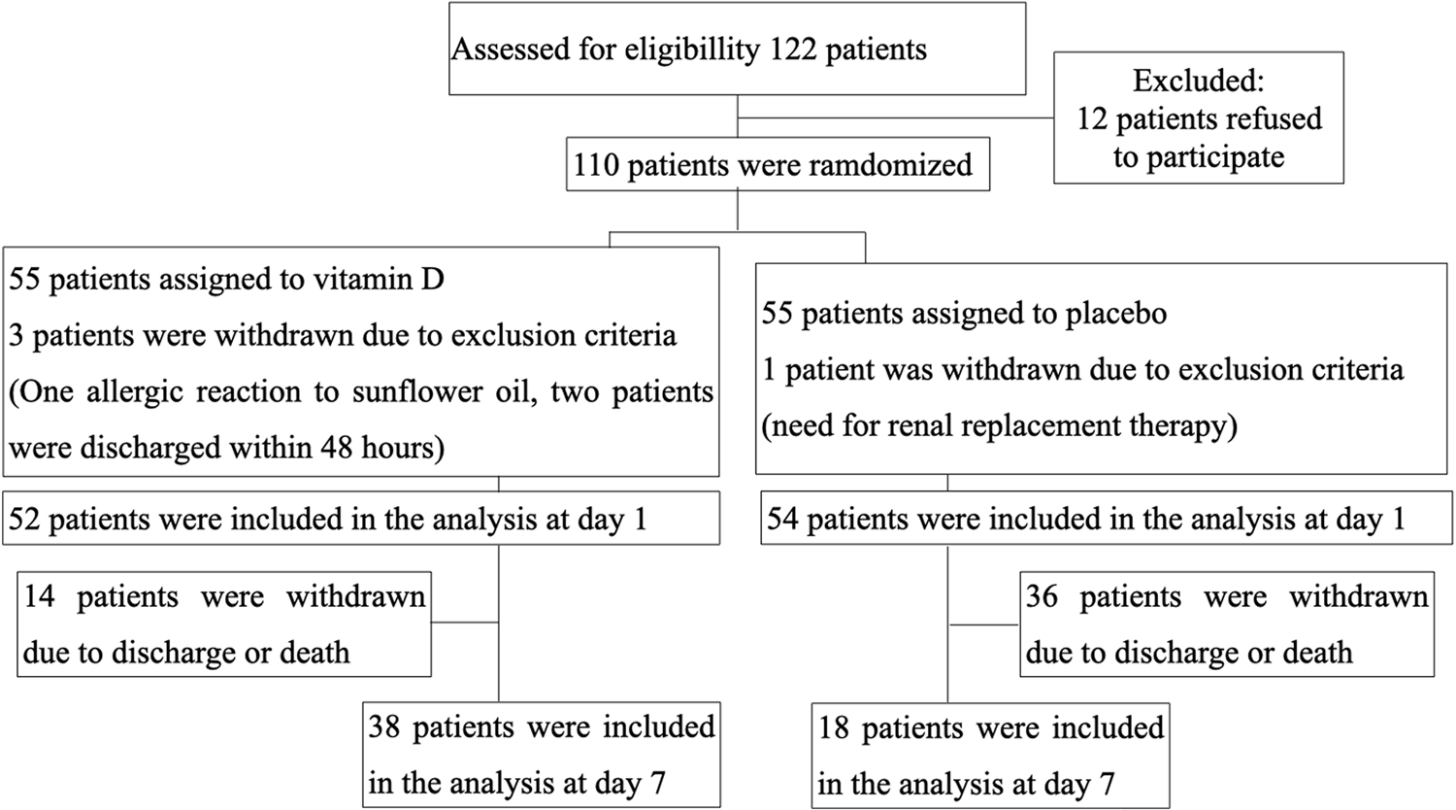
Flow diagram of the COVID-VIT trial.

One hundred and ten patients were enrolled in the study and randomized into an experimental group with vitamin D3 treatment or a placebo group without vitamin D3 treatment. The details are presented in the flow diagram (Fig. 2).

### Data presentation and statistics

Descriptive statistics were used for demographic, laboratory, and clinical prognostic factors related to COVID-19 in each treatment arm.

Continuous and categorical variables are presented as median (interquartile range, IQR) or n (%), as appropriate. Multiple comparisons were accounted for in the statistical analysis. Quantitative characteristics between the groups were compared using the Mann–Whitney *U* test. The dynamics of the quantitative characteristics in each group were performed using Wilcoxon’s test to assess within-group differences. χ^2^ tests (2 × 2) or Fisher’s exact test (if there were fewer than 10 observations) were performed to assess the significance of the differences between the characteristics according to the categorical variables. Spearman’s correlation analysis was performed to evaluate the relationship between the vitamin D levels and immunological parameters. Cox regression was used to estimate unadjusted and adjusted hazard ratios (HR) and 95% CIs for the probability of immunodeficiency and mortality. Differences were considered statistically significant at P < 0.05.

All statistical analyses were performed using SPSS for Mac v28 (IBM, USA). The pilot trial was conducted according to the Consolidated Standards of Reporting Trials (CONSORT) reporting guidelines.

Sample size calculation was carried out for the pilot study with 110 patients randomized in a proportion of 1:1. The sample size calculation was based on the proportion of participants treated with vitamin D3 that could meet the criteria for vitamin D deficiency, which was estimated as 25% (with 95% CIs), and the proportion of participants not treated with vitamin D3, which could be 50%. According to these assumptions, the estimated final sample size for our pilot clinical study was 55 patients in the arm of patients treated with vitamin D3 and 55 patients in the placebo group.

## Data Availability

The datasets are available from the corresponding author on reasonable request.

## Abbreviations

COVID-19: Coronavirus disease 2019
ICU: Intensive care unit
IL: Interleukin
ROC: Receiver operator characteristic
SARS-CoV-2: The severe acute respiratory syndrome coronavirus 2
25(OH)D: Serum 25-hydroxyvitamin D
NKT: Natural killer T
NK: Natural killer
NLR: Neutrophil-to-lymphocyte ratio
